# Effects of Stubble Height and Mulching on Wind Erosion in Maize–Peanut Intercropping Farmland

**DOI:** 10.3390/plants15203139

**Published:** 2026-10-14

**Authors:** Ping Zeng, Yuhang Wei, Jiaxin Chen, Yajun Su, Shuyao Wang, Naijia Wang, Yubo Meng, Chen Feng, Wuyan Xiang, Yunqing Wang, Meiling Zhang, Xining Gao, Liwei Wang

**Affiliations:** 1 College of Agronomy, Shenyang Agricultural University, Shenyang 110866, China; 2024220235@stu.syau.edu.cn (P.Z.); 2025220220@stu.syau.edu.cn (Y.W.); 2025220240@stu.syau.edu.cn (J.C.); 15834693296@163.com (Y.S.); 2025220236@stu.syau.edu.cn (S.W.); 13086857501naijiaw@163.com (N.W.); 2025240413@stu.syau.edu.cn (Y.W.); 18340610160@163.com (M.Z.); syaugxn@syau.edu.cn (X.G.); 2 Hebei Weather Modification Center, Shijiazhuang 050020, China; 15631903956@163.com; 3 Liaoning Academy of Agricultural Sciences, Shenyang 110161, China; sandyla570521@126.com (C.F.); xiangwy89@163.com (W.X.); 4 Shenyang Academy of Agricultural and Ecological Meteorology, Shenyang 110166, China

**Keywords:** farmland wind erosion, maize–peanut intercropping, stubble height, amount of wind erosion, wind erosion intensity

## Abstract

To clarify the effectiveness of different stubble heights and mulching patterns for wind erosion control in maize–peanut intercropping systems, this study was conducted in the black soil region of Northeast China using a combination of wind tunnel simulation and field wind erosion monitoring. Monocropped peanut bare land was used as the control (CK). Four wind tunnel treatments were established: maize stubble with mulching (WT-1), maize stubble without mulching (WT-2), and peanut strip with maize straw mulching (WT-3). Additionally, seven field treatments were established, comprising mulched and non-mulched plots at three stubble height levels (low, medium, and high), as well as a mulched peanut plot without stubble (T7). Wind erosion intensity and amount were systematically measured across all treatments. The results showed that: (1) Wind erosion intensity increased exponentially with wind speed (R^2^ = 0.90–0.96, *p* < 0.001). Over the full range of wind speeds, wind erosion intensity was consistently greater in CK and WT-2 than in WT-3 and WT-1. The three mulching treatments exhibited distinct protective characteristics across different wind speed intervals: rapid response of surface mulching at low wind speeds, enhanced wind erosion control of standing stubble at moderate wind speeds, and diminished stubble protection but sustained mulching advantage at high wind speeds. The WT-1 treatment maintained an anti-wind erosion efficiency of 70.15% even at the extreme wind speed of 18 m·s^−1^. (2) Field wind erosion amounts ranked as CK > T6 > T2 > T4 > T1 > T7 > T5 > T3. The medium stubble with mulching treatment (T3) showed a net dust deposition effect with a wind erosion amount of −0.35 g, demonstrating the optimal erosion reduction performance. (3) Standing stubble and surface mulching synergistically increased aerodynamic roughness by 22.86–394.44%, effectively reducing near-surface wind speed by 30.32–55.55%. Meanwhile, straw mulching reduced soil water evaporation, increased pre-sowing surface soil water content by 5.4–126.01%, and enhanced inter-particle cohesion, thereby effectively suppressing soil wind erosion. Therefore, after harvest in the maize–peanut intercropping system in the black soil region of Northeast China, the combined application of maize straw mulching on peanut strips (T7) and medium stubble (40–50 cm) with mulching on maize strips (T3) is recommended as an optimal technical strategy for wind erosion control in the study area and similar brown soil-dominated wind erosion-sensitive regions after autumn harvest.

## 1. Introduction

The black soil region of Northeast China serves as a crucial commercial grain production base and holds an irreplaceable strategic position in safeguarding national food security [1,2]. However, owing to a combination of natural environmental factors and anthropogenic influences, soil erosion in this region has become increasingly severe. In particular, the western part of the region, where wind erosion predominates, exhibits high erosion rates and strong surface winds, resulting in a much faster rate of black soil loss compared to the eastern part. The average thickness of the black soil layer in this area is merely 25 cm [3]. At present, the annual mean wind erosion modulus of farmlands in the northeast black soil region is 0.66 t·hm^−2^. Under persistent wind erosion, surface soil particles are continually removed, leading to serious degradation of black soil quality. This situation poses a grave threat to the productivity of arable land in Northeast China, and farmland soil wind erosion in this region has become increasingly prominent [4,5].

Soil wind erosion in farmland is governed by multiple interacting factors [6], among which near-surface wind speed and tillage practices are the primary controlling variables [7,8]. Wind serves as the direct driving force of soil wind erosion [9], which occurs only when wind speed exceeds the threshold velocity for particle entrainment [10,11]. From late winter to early spring, high winds and bare, loose surface soil frequently trigger wind erosion in the black soil region of Northeast China [12,13]. Tillage practices exert a pronounced regulatory effect on farmland wind erosion, and conservation tillage has been confirmed as an effective approach to mitigating this process [14,15]. Compared with conventional tillage, crop stubble retention and straw mulching—the core practices of conservation tillage—enhance aerodynamic roughness. Standing stubble, in particular, reduces the wind energy available for momentum transfer at the soil surface within the stubble layer, thereby elevating the threshold friction velocity required for the initiation of soil wind erosion [16]. In addition, surface straw mulching minimizes soil disturbance and reduces evaporative water loss, thereby maintaining soil water content. Soil water content is negatively correlated with wind erosion intensity; increased soil moisture elevates the threshold wind speed required for particle entrainment, thus suppressing soil wind erosion [17]. Although previous studies have demonstrated the benefits of stubble retention and straw mulching for wind erosion control in farmlands, most of them have treated stubble retention as a single, undifferentiated treatment without quantitative classification of stubble heights. In addition, the magnitude of protective effects under different stubble heights remains unclear. Furthermore, the interaction between stubble height and mulching method in intercropping systems, particularly maize–peanut intercropping, has not been systematically investigated. The dynamic changes in protective efficacy of different cover treatments during the bare period from post-harvest to pre-sowing also require further investigation [18,19,20].

Currently, wind erosion research primarily relies on field measurement methods and wind tunnel simulation techniques. Among field approaches, the wind erosion circle method is capable of capturing long-term erosion processes under natural environmental conditions. Wind tunnel simulation enables precise examination of erosion responses under controlled conditions [21,22]. Therefore, the integration of these two approaches not only combines the realism of field experiments with the controllability of wind tunnel tests, but also allows for mutual validation and complementarity, making it an effective strategy for in-depth investigation of farmland wind erosion mechanisms. However, current wind erosion research has largely relied on a single methodological approach, and comprehensive studies that integrate field in situ monitoring with wind tunnel simulation to systematically evaluate the wind erosion control effects of different cover treatments in maize–peanut intercropping farmland remain scarce.

To address these gaps, this study selected a typical maize–peanut intercropping farmland in the black soil region of Northeast China as the research object, focusing on the high-risk wind erosion period from winter to spring. By integrating field wind erosion circle monitoring with wind tunnel simulation experiments, we established three stubble height levels (low: 10–20 cm; medium: 40–50 cm; high: 60–70 cm) and mulching regimes (stubble with mulching vs. stubble without mulching). We systematically measured near-surface wind speed, wind erosion amount, and wind erosion intensity under different treatments to elucidate the synergistic regulatory effects of stubble height and mulching practices on soil wind erosion, and to identify the optimal technical strategy for wind erosion control. The findings are expected to provide a theoretical basis and technical support for wind erosion prevention and the integration of conservation tillage practices in the black soil region of Northeast China.

## 2. Study Area

**Figure 1:**
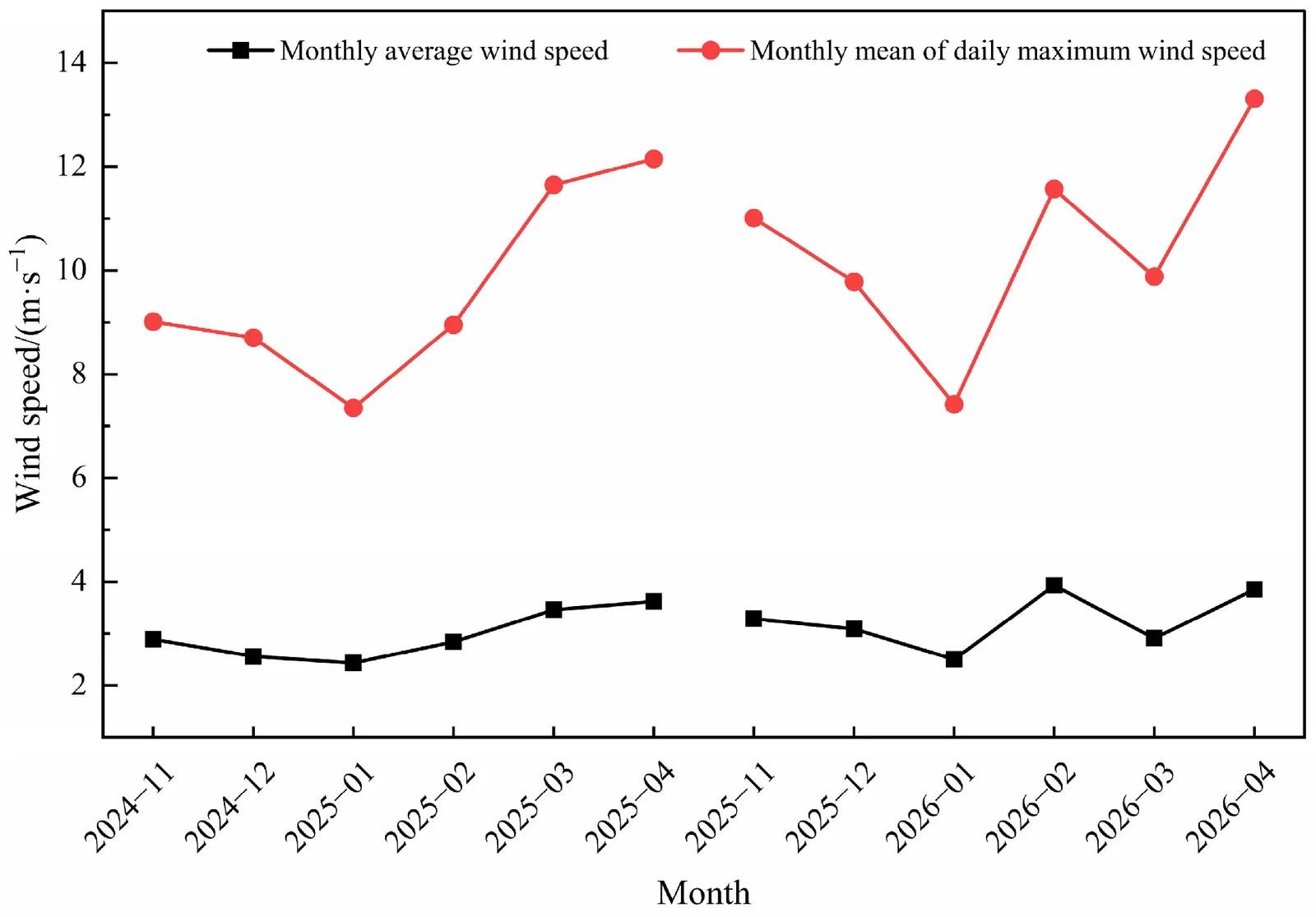
Monthly average wind speed and monthly average of daily extreme wind speed in the experimental area during the non-growing season from 2024 to 2026.

The study area is located in Changtu County, Tieling City, Liaoning Province, China (123°32′–124°26′ E, 42°33′–43°29′ N). The climate is characterized as a temperate continental monsoon climate, with four distinct seasons and concurrent rainfall and heat. The mean annual temperature is 9.6 °C, and the mean annual precipitation is 768 mm, with rainfall mainly concentrated in summer. The soil types are predominantly brown soil, meadow soil, and aeolian sandy soil. Wind is strong in spring, with prevailing southwesterly and northerly winds. Crops are generally sown in spring, and the land surface is left bare without cover after autumn harvest. The annual average wind speed is 2.8 m·s^−1^. During the non-growing season (from November to April of the following year) from 2024 to 2026, the mean wind speed in the study area was 3.1 m·s^−1^ (Figure 1), which is higher than the annual average. The monthly mean of daily extreme wind speeds generally followed the same trend as the monthly mean wind speeds. Comparison of the corresponding monthly wind speeds between the two years revealed a clear interannual difference. In February 2026, the mean wind speed surged to 3.93 m·s^−1^, the second highest peak during the non-growing season, then dropped to 2.91 m·s^−1^ in March, and rose again to 3.85 m·s^−1^ in April. In contrast, wind speeds during the same period in 2025 showed a steady increasing trend. Overall, the seasonal pattern was characterized by “high wind speeds in spring and low wind speeds in winter”. Two land types were included in the experimental area: monocropped peanut plowed land and maize–peanut intercropping land. Four plots were established for monocropped peanut bare land, and 28 plots were established for the intercropping system. Each plot measured 8 m in length and 4 rows in width, with a row spacing of 50 cm and a plant spacing of 30 cm. The maize-to-peanut row ratio was 4:4. The stubble density was maintained at the same level as the planting density, i.e., 60,000 plants hm^−2^. The basic physicochemical properties of the 0–20 cm plow layer prior to the experiment were as follows: pH 5.5, soil organic carbon (SOC) 8.65 g kg^−1^, total nitrogen (TN) 0.92 g kg^−1^, total phosphorus (TP) 0.40 g kg^−1^, total potassium (TK) 21.16 g kg^−1^, alkali-hydrolyzable nitrogen (AN) 107.1 mg kg^−1^, available phosphorus (AP) 28.3 mg kg^−1^, available potassium (AK) 166.6 mg kg^−1^, and bulk density (BD) 1.40 g cm^−3^.

## 3. Materials and Methods

### 3.1. Wind Tunnel Simulation Experiment

#### 3.1.1. Experimental Sample Collection and Processing

After harvest in the maize–peanut intercropping system, in the black soil region of Northeast China under the influence of mechanized harvesting operations, the maize strips predominantly retain standing stubble. The remaining maize stalks, after being mechanically chopped, can either be directly returned to the maize strips or broadcast as surface mulch over the adjacent harvested peanut bare land, thereby forming differentiated surface cover patterns within the intercropping system. However, it is difficult to establish an ideal bare-land control within the intercropping system by removing all surface residues. On the one hand, removing maize root stubble is inconsistent with local conventional harvesting practices, significantly increases operational costs, and severely disrupts topsoil structure. On the other hand, the adjacent peanut strips are inevitably affected by straw residue from the maize strips, as well as by other natural and anthropogenic disturbances, making it impossible to maintain a stable bare-land state over time.

Therefore, in this study, monocropped peanut harvested bare land, which shares similar tillage conditions with the intercropped peanut strips, was selected as the control (CK). Three additional treatments were used: maize stubble with mulching (WT-1), maize stubble without mulching (WT-2), and maize–straw mulching on peanut strips (WT-3). The detailed treatment schemes are presented in Table 1. To clarify the wind erosion control effects and their stability under different wind speeds for various mulching treatments, undisturbed soil samples were collected from representative plots in the study area after the autumn harvest in November 2025. Each treatment was replicated three times (*n* = 3), with all replicates arranged in a completely randomized design in the field. The undisturbed soil samples measured 75 cm in length, 30 cm in width, and 8 cm in height, with a mass of approximately 20 kg. Sampling was conducted using stainless steel rectangular frames. Each frame was carefully pressed into the soil in the field and then excavated as an intact block, with minimal disturbance to surface residues and mulch. During sampling, photographs were taken to record the surface conditions of each treatment, and quantitative measurements of straw mulch cover were also performed. This ensured that the surface conditions of the soil samples used in the wind tunnel experiment were consistent with the field-measured data. After collection, the samples were air-dried indoors prior to wind tunnel simulation experiments. For the stubble and mulch materials damaged during sample collection, they were restored using maize stubble and stalks collected synchronously from the same plots, following the residue conditions determined from field measurements, to ensure that the surface conditions in the laboratory were essentially consistent with those in the field.

#### 3.1.2. Experimental Instruments and Methods

The wind tunnel simulation experiment was conducted in November 2025 at the Soil Composite Erosion Laboratory of Shenyang Agricultural University. The wind tunnel is a direct-blowing type, with a test section of 6 m in length, 1 m in width, and 0.6 m in height. The wind speed is continuously adjustable within the range of 4–30 m·s^−1^ with stable airflow. The boundary layer thickness of the wind tunnel is 0.28 m, which fully meets the requirements for wind erosion simulation. A pre-cut opening for the erosion tray was reserved on the floor of the test section, measuring 0.75 m in length and 0.30 m in width, with an area of 0.225 m^2^. The lower part is hollow, and a set of freely adjustable supports is used to hold the wind erosion samples. During the experiment, the soil samples were embedded in the erosion tray, with the soil surface layer approximately 1 cm thick, which is much smaller than the boundary layer thickness of the wind tunnel.

In the wind tunnel experiment, the minimum wind speed at the center of the test section was set to 6 m·s^−1^, and the maximum wind speed was set to 18 m·s^−1^. Each treatment underwent two rounds of experiments, with each round consisting of six wind speeds: 6, 8, 10, 12, 14, and 18 m·s^−1^. The corresponding exposure durations were 15, 12, 10, 8, 5, and 3 min, respectively. The aim was to investigate the wind erosion intensity of different mulching treatments under varying wind speeds.

Based on the wind tunnel simulation data, the wind erosion intensity of each mulching treatment under different wind speeds after the autumn harvest was calculated using Equation (1).

(1) $$Q_{t}=\frac{W}{S\times T}$$

where *Q*_t_: wind erosion intensity (g·m^−2^·h^−1^); *W*: mass difference of the soil sample before and after wind erosion (g); *S*: surface area of the test sample (m^2^); and *T*: duration of wind erosion (h).

Using the wind erosion intensity of the bare peanut field after harvest under monocropping at each wind speed as the reference, the anti-wind erosion efficiency of each mulching treatment was calculated as the ratio of the difference between the treatment and reference intensities to the reference intensity, expressed as a percentage. The calculation formula is given as follows:

(2) $$\eta =\frac{Q_{1}-Q_{2}}{Q_{1}}\times 100\%$$

where *η*: anti-wind erosion efficiency of each mulching treatment at each wind speed (%); *Q*_1_: wind erosion intensity of the bare peanut field after harvest under monocropping (g·m^−2^·h^−1^); and *Q*_2_: wind erosion intensity of each mulching treatment (g·m^−2^·h^−1^).

### 3.2. Field Wind Erosion Cylinder Experiment

The field wind erosion cylinder experiment was conducted in the study area. The plots included monocropped peanut plowed land and maize–peanut intercropping land. Monocropped peanut plowed land served as the control (CK). In the intercropping plots, at maize harvest, the header height of the combine harvester was adjusted. This created three stubble heights: low, medium, and high. In addition, seven treatments with different stubble heights and mulching practices were established. Each treatment had four replicates (*n* = 4), with all replicates arranged in a completely randomized design in the field. Detailed treatment information is provided in Table 2. Due to inherent operational variability in field machinery, the actual stubble heights of each designed stubble level, as measured in the field, fluctuated within a certain range. The roughness length was derived from wind profile measurements at the plot scale, thus reflecting the integrated effect of the stubble height range within each plot and serving as a valid representative value of the plot-scale surface roughness.

In this experiment, the field wind erosion cylinder method was employed to determine changes in soil wind erosion amount. In November 2025, prior to winter soil freezing, an appropriate amount of farmland soil was collected, measured for soil water content, weighed, and then placed into wind erosion cylinders with an inner diameter of 22 cm and a height of 3 cm. The upper surface of the cylinders was leveled with the ground surface, and the lower surface was in full contact with the underlying soil, ensuring that the soil within the cylinders formed an integrated continuum with the natural field soil. The cylinders were then retrieved in April 2026, before spring sowing. Upon retrieval, the wet weight and water content of the soil in each cylinder were determined. The soil wind erosion amount for each mulching treatment was subsequently calculated using Equation (3).

*W* = *W*_1_ × (1 − *X*_1_) − *W*_2_ × (1 − *X*_2_) (3)

where *W*: total soil wind erosion amount over the entire erosion period (g); *W*_1_: wet weight of the soil placed into the wind erosion cylinder (g); *W*_2_: wet weight of the soil at the time of cylinder retrieval (g); *X*_1_: gravimetric soil water content at the time of placement into the cylinder (%); and *X*_2_: gravimetric soil water content at the time of cylinder retrieval (%).

In the experiment, a handheld anemometer was used to measure the instantaneous wind speeds at heights of 0.05 m and 0.5 m of different mulching treatments and the control group during two periods: after autumn harvest and before spring sowing. A digital multifunctional anemometer was used to measure wind speed data at different heights for different mulching treatments after autumn harvest and before spring sowing. The wind speed readings for all treatments were collected synchronously within the same time period to avoid interference with comparisons among treatments caused by changes in meteorological conditions over time. During measurement, the wind wheel was perpendicular to the prevailing wind direction, and the anemometer wind wheel heights were set at 0.05 m and 2 m. Starting at 14:00, measurements were taken continuously for 15 min, with a data recording interval of 5 s. The aerodynamic roughness for each mulching treatment was then calculated using Equation (4) [23]. It should be noted that, owing to the limitations of the experimental conditions, this study estimated the aerodynamic surface roughness based solely on wind speeds at two heights, 0.05 m and 2 m. Therefore, the results are mainly intended for relative comparisons among different mulching treatments, and their absolute values should be interpreted with caution.

(4) $$\mathrm{lg}R_{0}=\frac{\left(V_{1}\mathrm{lg}Z_{2}-V_{2}\mathrm{lg}Z_{1}\right)}{\left(V_{1}-V_{2}\right)}$$

where *R*_0_: aerodynamic roughness, which reflects the attenuating effect of the surface on wind speed and represents the height at which the mean wind speed theoretically reduces to zero; it depends on soil texture and cover type (m). A larger *R*_0_ indicates a rougher soil surface and a more pronounced weakening effect on near-surface wind speed; *Z*_1_ and *Z*_2_ are the measurement heights (m). In the experiment, *Z*_1_ = 0.05 m, within the roughness sublayer and sensitive to surface cover changes, while *Z*_2_ = 2 m, above the farmland boundary layer, avoids canopy and obstacle disturbances and represents free-flow conditions; *V*_1_: wind speed at height *Z*_1_ (m·s^−1^); and *V*_2_: wind speed at height *Z*_2_ (m·s^−1^).

### 3.3. Data Processing

The experimental data were compiled using Microsoft Excel (v2609) and subsequently analyzed and visualized with Origin2026 software. Significance tests for all measured variables were performed using the least significant difference (LSD) method, with the significance level set at *p* < 0.05. Regression analysis was performed using a stepwise comparison approach to determine the optimal fitting function describing the relationship between wind erosion intensity and wind velocity for each treatment. Four candidate models were compared for each treatment: linear function (*Y* = a + bx), logarithmic function (*Y* = a + b ln(x − c)), quadratic polynomial (*Y* = a + bx + cx^2^), and exponential function (*Y* = ae^bx^). The optimal model for each treatment was selected based on the highest coefficient of determination (*R*^2^) and the lowest root mean square error (RMSE) as the primary criteria, combined with the randomness of the residual distribution for comprehensive evaluation. All data are presented as mean ± standard error (mean ± SE).

## 4. Results and Analysis

### 4.1. Effects of Different Mulching Treatments on Soil Wind Erosion Intensity

#### 4.1.1. Relationship Between Soil Wind Erosion Intensity and Wind Speed Under Different Mulching Treatments

**Figure 2:**
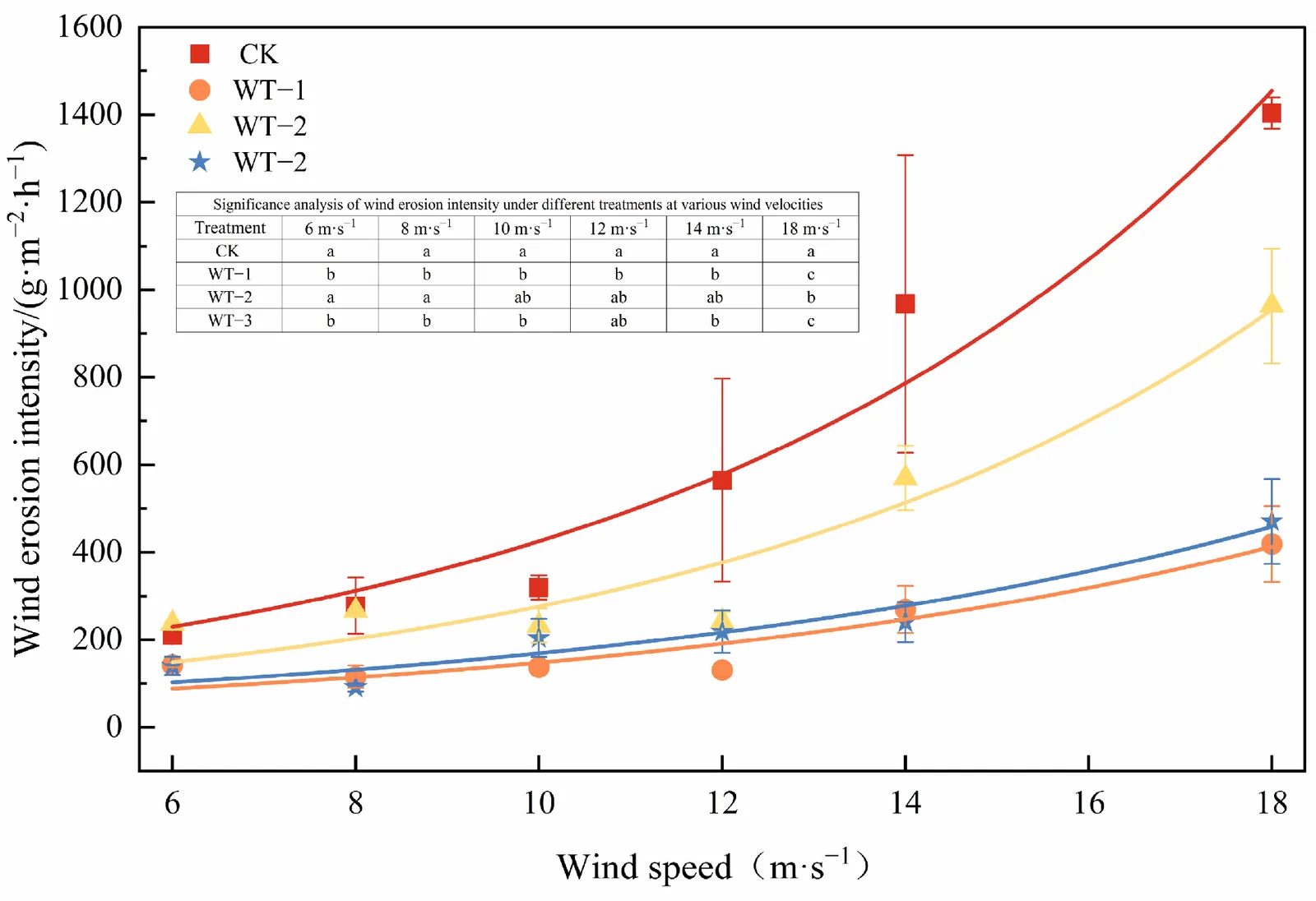
Relationship curves between soil wind erosion intensity and wind velocity under different treatments. **Note:** Different lowercase letters indicate significant differences among treatments based on the letter-marking method (*p* < 0.05). The same note applies below.

Under wind tunnel conditions that closely simulated the field environment, wind erosion intensity increased exponentially with increasing wind speed for all treatments (Figure 2), with coefficients of determination (*R*^2^) ranging from 0.90 to 0.96 and all regression models being statistically significant at *p* < 0.001 (Table 3). Among the treatments, CK and WT-2 exhibited faster response rates of wind erosion intensity to wind speed. At wind speeds of 6–8 m·s^−1^, wind erosion intensity in the CK and WT-2 treatments was significantly greater than that in the WT-1 and WT-3 treatments. At 10 m·s^−1^, the WT-1 and WT-3 treatments showed significantly lower wind erosion intensity than the CK treatment, but no significant difference was observed relative to the WT-2 treatment. At 12 m·s^−1^, the disparities among treatments began to widen, with wind erosion intensity ranking as CK > WT-2 > WT-3 > WT-1. At 14 m·s^−1^, CK and WT-2 showed sharp increases in wind erosion intensity. Specifically, the CK treatment reached 967.54 g·m^−2^·h^−1^, which was significantly higher than that of the WT-1 and WT-3 treatments, being 3.59 and 4.03 times greater than the WT-1 (269.88 g·m^−2^·h^−1^) and WT-3 (240.20 g·m^−2^·h^−1^) treatments, respectively; WT-2 surged to 570.19 g·m^−2^·h^−1^, representing a 4.36 times increase compared with its value at 12 m·s^−1^. At the extreme wind speed of 18 m·s^−1^, the CK treatment exhibited a wind erosion intensity of 1403.78 g·m^−2^·h^−1^, which was significantly higher than all other treatments, being 3.35 and 2.98 times greater than that of the WT-1 (418.98 g·m^−2^·h^−1^) and WT-3 (470.89 g·m^−2^·h^−1^) treatments, respectively. The WT-2 treatment reached 963.37 g·m^−2^·h^−1^.

#### 4.1.2. Variations in Anti-Wind Erosion Efficiency with Wind Speed Under Three Mulching Treatments

**Figure 3:**
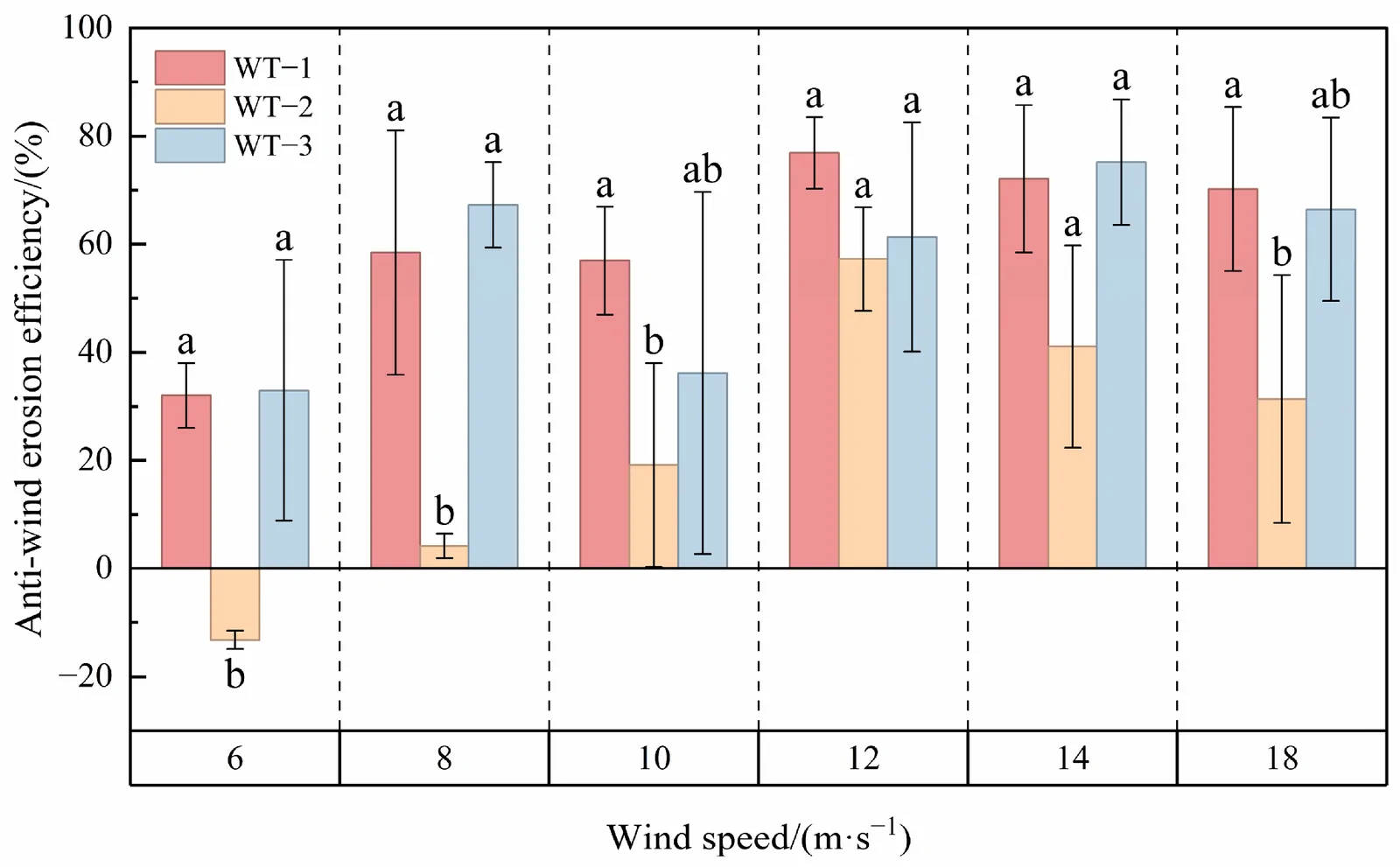
Relationship of anti-wind erosion efficiency with wind velocity under three mulching treatments. **Note:** Different lowercase letters in the figure indicate significant differences in anti-wind erosion efficiency among the three mulching treatments at the same wind velocity (*p* < 0.05).

The anti-wind erosion efficiencies of the three mulching treatments at different wind speeds (Figure 3) further revealed significant differences among treatments. At 6 m·s^−1^, WT-2 showed an anti-wind erosion efficiency of −13.2%, significantly lower than the other treatments, indicating that it exacerbated wind erosion; by contrast, both WT-1 and WT-3 achieved 32.03% efficiency and maintained relatively high values throughout the 6–18 m·s^−1^ wind speed range. At 8 m·s^−1^, WT-1 and WT-3 exhibited significantly higher efficiencies than WT-2, which recorded only 4.14%. At 10 m·s^−1^, the differences among treatments narrowed; WT-1 maintained the best wind erosion control performance (56.96%), with both WT-1 and WT-3 being significantly more efficient than WT-2. In the 12–14 m·s^−1^ range, no significant differences were detected among treatments, although WT-1 and WT-3 consistently outperformed WT-2; WT-1 reached 76.87% at 12 m·s^−1^, and WT-3 reached 75.17% at 14 m·s^−1^. At the extreme wind speed of 18 m·s^−1^, WT-1 exhibited the highest and most stable anti-wind erosion efficiency (70.15%), significantly greater than that of WT-2, while WT-3 achieved 66.46%.

### 4.2. Field Wind Erosion Cylinder Experiment

#### 4.2.1. Effects of Different Treatments on Field Instantaneous Wind Speed

**Figure 4:**
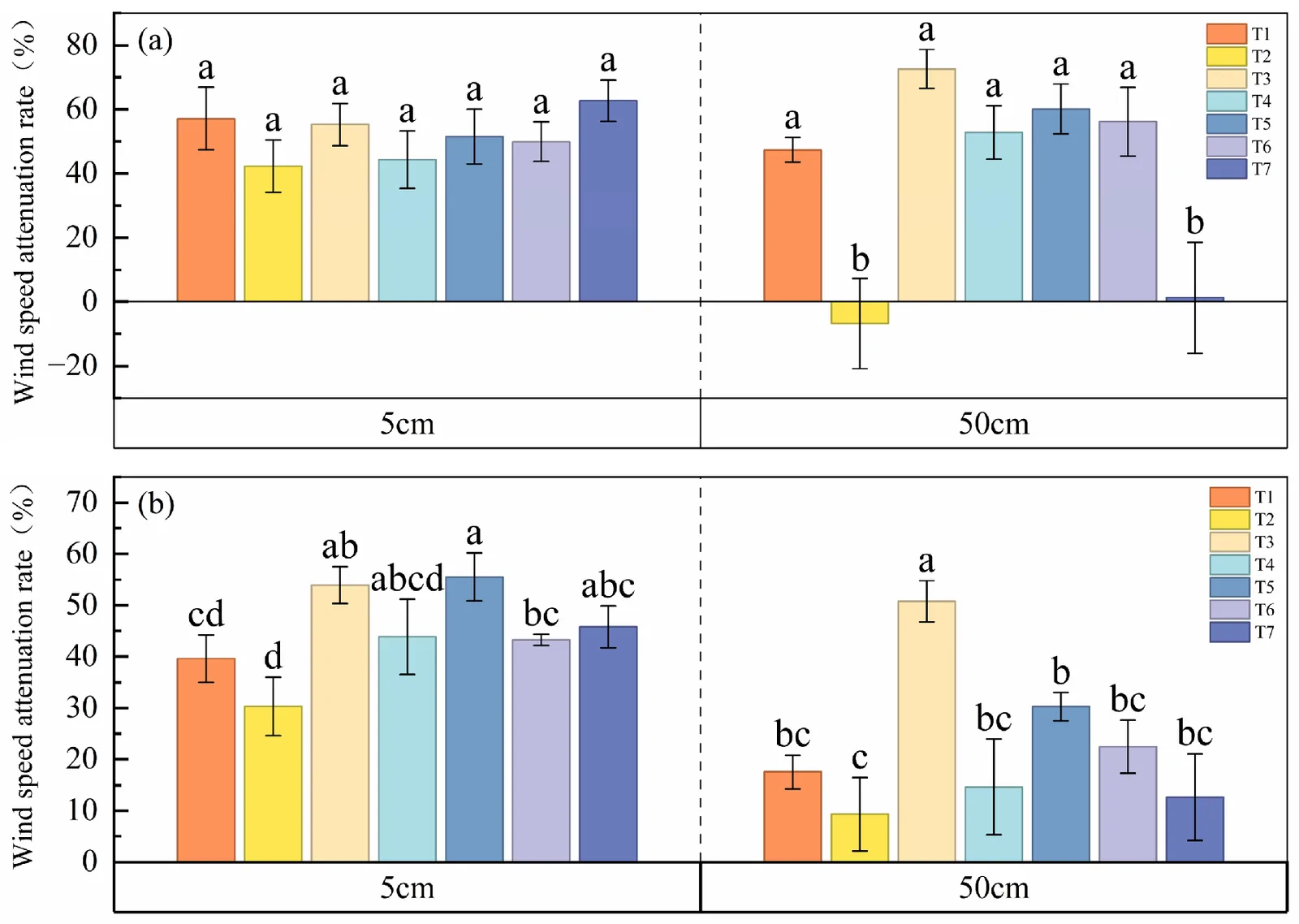
Wind speed attenuation rates at different heights for different mulching treatments after autumn harvest (November 2025) (**a**) and before spring sowing (April 2026) (**b**). **Note:** Different lowercase letters indicate significant differences among mulching treatments at the same height (*p* < 0.05).

During the two key farmland management periods (after autumn harvest and before spring sowing), significant differences in wind speed attenuation rates were observed among treatments (T1–T7) relative to CK at heights of 5 cm and 50 cm. After autumn harvest (Figure 4a), all treatments generally exhibited strong wind speed attenuation at 5 cm height, with attenuation rates ranking as T7 > T1 > T3 > T5 > T6 > T4 > T2; T7, T1, T3, and T5 showed attenuation rates of 62.65%, 57.14%, 55.26%, and 51.51%, respectively, with no significant differences among them. At 50 cm height, the ranking was T3 > T5 > T4 > T6 > T1 > T7 > T2; T2 exhibited a negative attenuation rate (wind speed enhancement relative to CK), and T7 showed only 1.27% attenuation, both being significantly lower than the other treatments. Before spring sowing (Figure 4b), all treatments maintained relatively high attenuation at 5 cm, ranking as T5 > T3 > T7 > T4 > T6 > T1 > T2, with T5 (55.55%) significantly higher than T1, T2, and T6. At 50 cm, negative attenuation disappeared, although attenuation rates declined to varying degrees compared with those after autumn harvest; the ranking was T3 > T5 > T6 > T1 > T4 > T7 > T2, with T3 (50.77%) significantly higher than all other treatments and T2 the lowest (9.35%). Overall, at both 5 cm and 50 cm heights, stubble-retained plots with mulching consistently exhibited higher attenuation rates than their corresponding stubble-retained plots without mulching. Notably, before spring sowing, the medium-stubble with mulching treatment (T3) showed significantly higher attenuation rates at both heights than its no-mulching counterpart (T4).

To further quantify the response characteristics of wind speed attenuation rate (*Y*) of each mulching treatment to near-surface wind speed (*V*), this study conducted regression analysis on the relationship between the two variables using field wind speed data (Table 4). The results showed that the coefficients of determination (*R*^2^) for all treatments ranged from 0.93 to 0.99. Except for the T7 treatment, the regression equations of all mulching treatments reached extremely significant or significant levels (*p* < 0.01), indicating a good fitting effect. From the regression equations, the wind speed attenuation rates of different mulching treatments exhibited distinct response patterns with changing wind speed. The wind speed attenuation rate of the T1 treatment first increased and then decreased with increasing wind speed, with an optimal wind speed range, and declined when wind speed exceeded 2.60 m·s^−1^. The wind speed attenuation rates of T2–T6 treatments showed logarithmic growth with increasing near-surface wind speed (*Y* = a + b·ln(*V* − c)), with R^2^ values of 0.96–0.99, indicating that the attenuation rate increased rapidly at lower wind speeds and gradually leveled off after reaching a certain threshold, with different wind speed response thresholds and sensitivity ranges among treatments. Among them, the threshold wind speed for the T3 treatment was 0.96 m·s^−1^, and its wind speed attenuation rate remained consistently above −13.72% within its domain, enabling wind speed attenuation at extremely low wind speeds. The T5 treatment was the most sensitive to wind speed changes, with its attenuation rate increasing rapidly with increasing wind speed; however, its threshold wind speed was high, requiring at least 2.03 m·s^−1^ for surface residue and mulching to begin exerting a positive attenuation effect on near-surface wind speed, and this threshold value was the highest among T2-T6 treatments. The regression equation for the T7 treatment exhibited a piecewise function pattern: at wind speeds below 3.5 m·s^−1^, the wind speed attenuation rate remained stable at approximately 67.55%; however, once wind speed exceeded 3.5 m·s^−1^, the attenuation rate declined sharply, reaching its lowest value at 4.57 m·s^−1^, and then gradually recovered, returning to positive attenuation at 6.29 m·s^−1^.

#### 4.2.2. Effects of Different Treatments on Surface Soil Water Content

**Figure 5:**
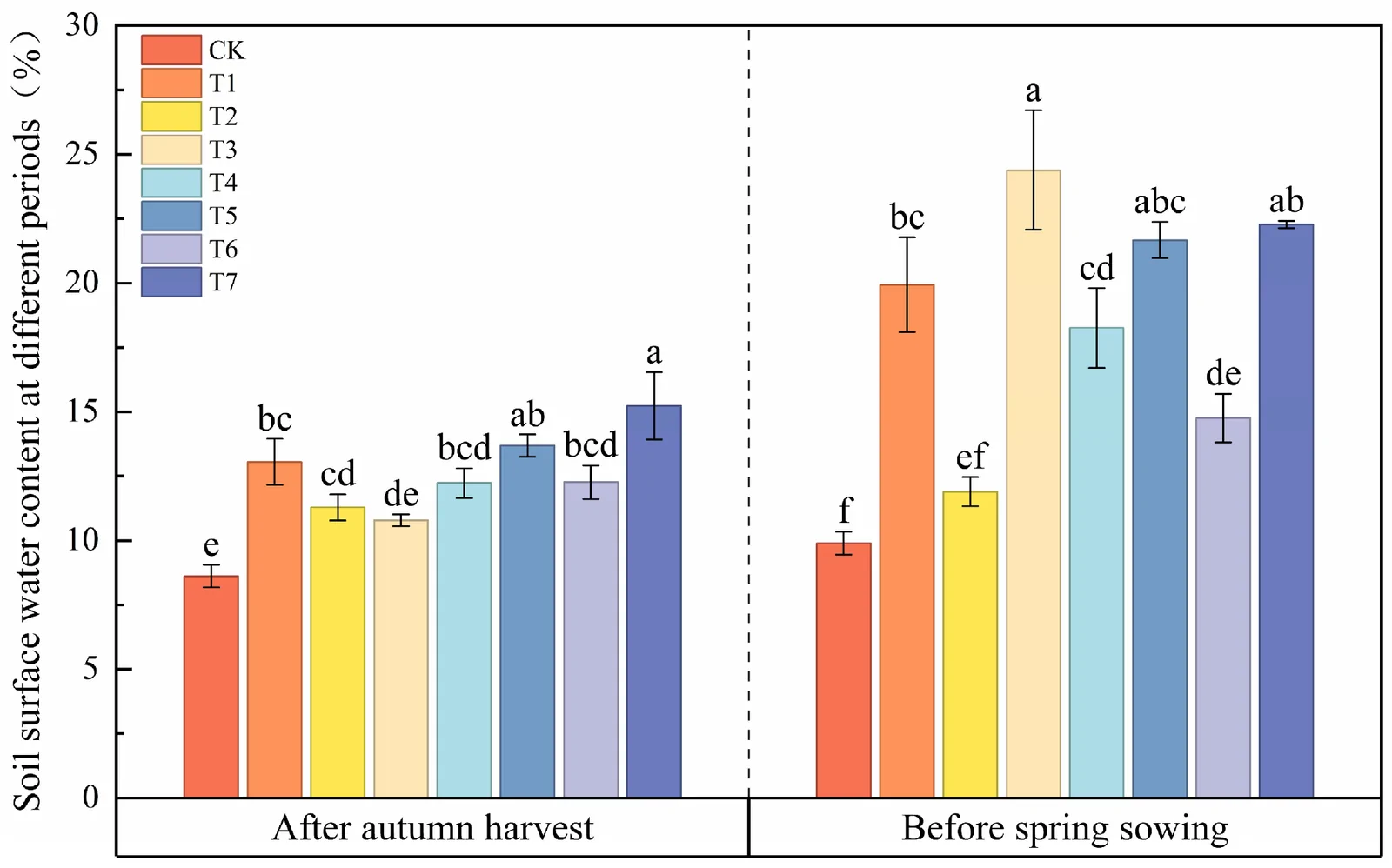
Surface soil water content of different mulching treatments at different periods. **Note:** Different lowercase letters indicate significant differences among mulching treatments at the same height (*p* < 0.05).

Significant differences in surface soil water content were observed among mulching treatments at different periods (Figure 5). After autumn harvest, surface soil water content ranked as T7 > T5 > T1 > T6 > T4 > T2 > T3 > CK; T7 (15.23%) was significantly higher than all other treatments except T5, while CK (8.62%) was significantly lower than all other treatments except T2 and T6. Before spring sowing, surface soil water content increased to varying degrees compared with that after autumn harvest for all treatments, ranking as T3 > T7 > T5 > T1 > T4 > T6 > T2 > CK; CK (9.90%) was significantly lower than all other treatments except T2. Under different stubble heights, stubble-retained plots with mulching consistently exhibited significantly higher surface soil water content than their corresponding stubble-retained plots without mulching. Notably, T3 (medium stubble with mulching) showed a surface soil water content of 24.39%, which was significantly higher than all other treatments except T5 (21.67%) and T7 (22.27%).

#### 4.2.3. Aerodynamic Roughness Length of Different Treatments at Various Periods

**Figure 6:**
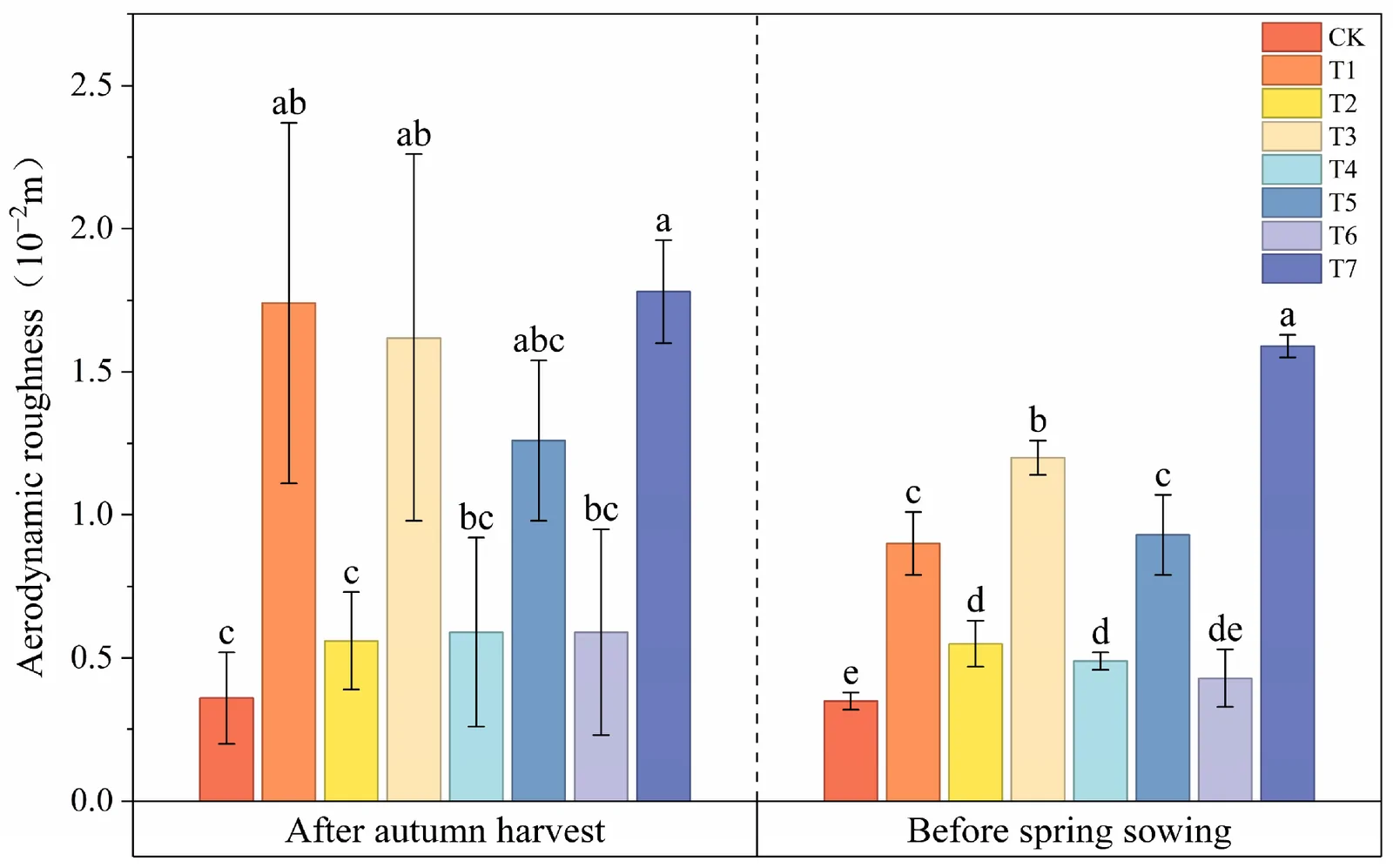
Aerodynamic roughness of different mulching treatments at different periods. **Note:** Different lowercase letters indicate significant differences among mulching treatments at the same height (*p* < 0.05).

Significant differences in aerodynamic roughness were observed among different mulching treatments after autumn harvest and before spring sowing (Figure 6). After autumn harvest, the aerodynamic roughness length of treatments ranked as T7 > T1 > T3 > T5 > T4 > T6 > T2 > CK. The aerodynamic roughness length of T7 was significantly higher than that of CK, T2, T4, and T6, while CK was significantly lower than T7, T1, and T3. Under different stubble heights, stubble retention with mulching treatments exhibited a higher aerodynamic roughness length than the corresponding stubble retention without mulching treatments, though the differences were not statistically significant. Moreover, the aerodynamic roughness length of mulched plots with different stubble heights tended to decrease with increasing stubble height, but the differences among groups were also not significant. Before spring sowing, the aerodynamic roughness length of treatments ranked as T7 > T3 > T1 > T4 > T6 > T5 > CK > T2. The aerodynamic roughness length of T7 was significantly higher than that of all other treatments, while CK was significantly lower than T1, T2, T3, and T7. Under different stubble heights, low- and medium-stubble retention with mulching treatments had a significantly higher aerodynamic roughness length than the corresponding stubble retention without mulching treatments. For the high-stubble treatments, T5 (high stubble with mulching) exhibited a slightly lower aerodynamic roughness length than T6 (high stubble without mulching), though the difference was not significant. Among them, T3 (medium stubble with mulching) had a significantly higher aerodynamic roughness length than T1 (low stubble with mulching) and T5 (high stubble with mulching).

#### 4.2.4. Soil Wind Erosion Amount Under Different Mulching Treatments

**Figure 7:**
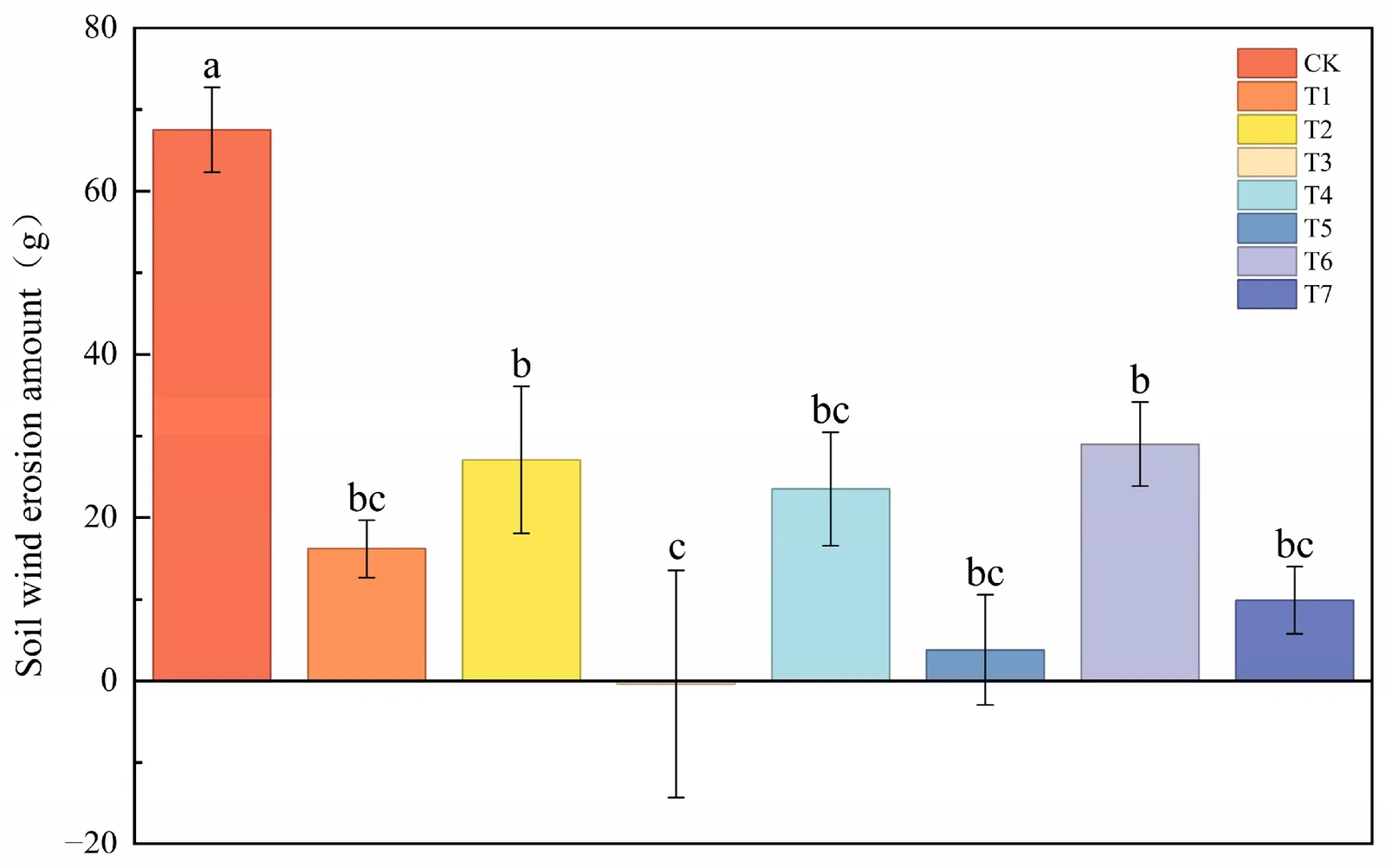
Soil wind erosion amount under different mulching treatments. **Note:** Different lowercase letters indicate significant differences among mulching treatments at the same height (*p* < 0.05).

Significant differences in soil wind erosion amount were observed among different mulching treatments (Figure 7), which were closely related to surface cover conditions. Soil wind erosion amount ranked as CK > T6 > T2 > T4 > T1 > T7 > T5 > T3. CK (67.53 g) was significantly higher than all mulching treatments. Notably, T3 (medium stubble with mulching) showed a negative wind erosion amount of −0.35 g, indicating net wind deposition, and was significantly lower than CK, T2, and T6. Compared with CK, the reductions in wind erosion amount for T1, T2, T4, T5, T6, and T7 were 76.04%, 59.89%, 65.15%, 94.37%, 57.06%, and 85.38%, respectively. Under different stubble heights, stubble-retained plots with mulching consistently exhibited lower wind erosion amounts than their corresponding stubble-retained plots without mulching, although the differences were not statistically significant. Among the mulched treatments, T3 (medium-height maize stubble with mulch) recorded the lowest wind erosion amount, at −0.35 g, representing a net deposition. This suggests that the amount of airborne particles trapped by the treatment exceeded the soil loss from the plot.

#### 4.2.5. Correlating Soil Wind Erosion with Temporal Variations in Wind Erosion Factors

**Figure 8:**
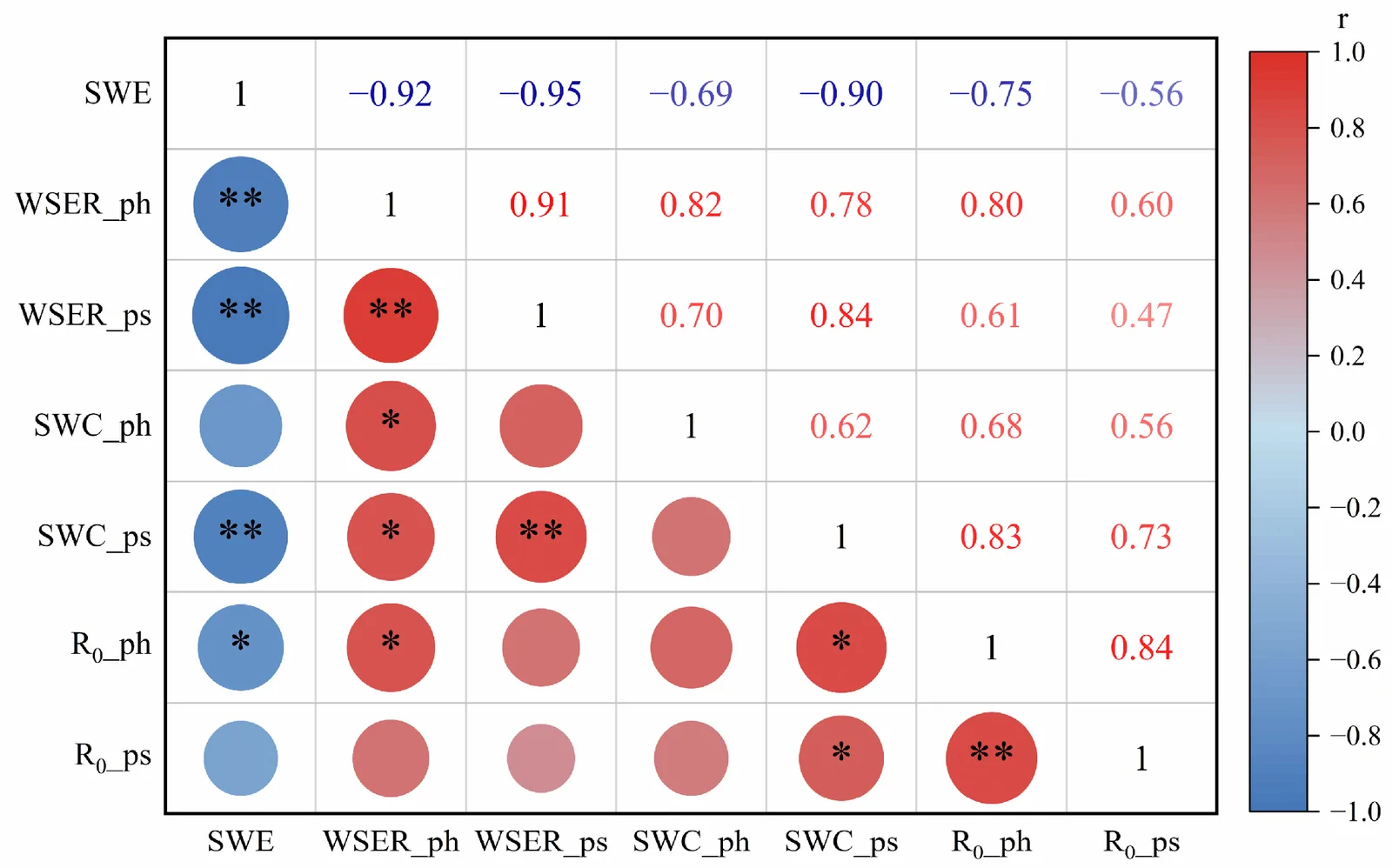
Correlation coefficients. **Note:** r is the correlation coefficient; SWE, soil wind erosion amount (*n* = 32); WSER_ph, wind speed attenuation rate above stubble (5 cm) after autumn harvest (*n* = 282); WSER_ps, wind speed attenuation rate above stubble (5 cm) before spring sowing (*n* = 348); SWC_ph, surface soil water content after autumn harvest (*n* = 32); SWC_ps, surface soil water content before spring sowing (*n* = 32); R0_ph, aerodynamic roughness after autumn harvest (*n* = 3677); R0_ps, aerodynamic roughness before spring sowing (*n* = 3606). * *p* < 0.05; ** *p* < 0.01.

The correlation analysis between soil wind erosion amount (SWE) and various wind erosion factors is presented in Figure 8. SWE was negatively correlated with all wind erosion factors. Specifically, it was significantly negatively correlated with the near-surface (5 cm) wind speed attenuation rate after autumn harvest (r = −0.92, *p* < 0.01), the near-surface (5 cm) wind speed attenuation rate before spring sowing (r = −0.95, *p* < 0.01), and the surface soil water content before spring sowing (r = −0.90, *p* < 0.01), and significantly correlated with the aerodynamic roughness length after autumn harvest (r = −0.75, *p* < 0.05). Among them, the wind speed attenuation rate at 5 cm above the ground before spring sowing exhibited the highest absolute correlation coefficient with SWE, indicating that this factor possesses the strongest explanatory power for wind erosion variability and represents a key link in regulating wind erosion. The near-surface wind speed attenuation rate after autumn harvest was significantly positively correlated with that before spring sowing (r = 0.91, *p* < 0.01), and significantly positively correlated with the surface soil water content after autumn harvest (r = 0.82, *p* < 0.05), the surface soil water content before spring sowing (r = 0.78, *p* < 0.05), and the aerodynamic roughness length after autumn harvest (r = 0.72, *p* < 0.05). The near-surface wind speed attenuation rate before spring sowing was significantly positively correlated with the surface soil water content before spring sowing (r = 0.80, *p* < 0.01). The aerodynamic roughness length after autumn harvest was significantly positively correlated with that before spring sowing (r = 0.83, *p* < 0.05) and with the surface soil water content before spring sowing (r = 0.73, *p* < 0.05). The aerodynamic roughness length before spring sowing was significantly positively correlated with that after autumn harvest (r = 0.84, *p* < 0.01).

## 5. Discussion

### 5.1. Response of Wind Erosion Intensity and Anti-Wind Erosion Efficiency to Wind Speed Under Different Mulching Treatments

The results of this study further confirm the general pattern that farmland wind erosion intensity increases exponentially with increasing wind speed. Once the wind speed exceeds the threshold for saltation initiation, coarser soil particles are lifted, leading to a sharp increase in erosion intensity [24,25]. Across the entire wind speed range, wind erosion intensity under different mulching treatments was generally higher for CK and WT-2 than for WT-1 and WT-3, with notable differences in both the initial coefficients and the growth rates of the exponential curves among treatments, which are closely related to the regulation of surface conditions by the mulching treatments. The bare CK treatment lacked effective roughness elements, allowing near-surface wind to act directly on soil particles. This resulted in a low threshold wind velocity for erosion initiation, high particle entrainment efficiency, and the fastest response of wind erosion intensity to increasing wind speed, with a sharp increase occurring at wind speeds above 14 m·s^−1^ [26]. In contrast, WT-1 established a three-dimensional erosion control system through the combination of standing stubble and surface mulching. The standing stubble acted as roughness elements that effectively raised the saltation layer, increased aerodynamic roughness, directly dissipated near-surface wind momentum, and intercepted saltating particles. Meanwhile, the surface mulch protected loose topsoil particles from direct wind shear. The synergistic effect of these two components—stubble first reducing wind speed, followed by further dissipation of residual wind energy by the mulch layer—created a cascading attenuation mechanism that prevented wind momentum from concentrating on the soil surface. This enabled WT-1 to maintain consistently high and stable anti-wind erosion efficiency across the entire 6–18 m·s^−1^ wind speed range [27].

Although both WT-1 and WT-2 involved stubble retention, WT-2 exhibited unstable wind erosion control. At low wind speed (6 m·s^−1^), WT-2 even showed a negative effect (i.e., exacerbated erosion), which may be attributed to the small sample surface area and low cover in the wind tunnel experiment, where standing stubble alone provided limited wind speed attenuation [28]. Alternatively, localized obstruction by the stubble may have created narrow channels or vortex zones that enhanced local wind speed, thereby intensifying erosion at low wind speeds—a mechanism requiring further investigation [29]. At moderate wind speeds (8–12 m·s^−1^), the aerodynamic effect of standing stubble became somewhat effective; however, due to the absence of surface mulch as a secondary mechanism for continuous momentum dissipation, the growth rate of erosion intensity (0.15) for WT-2 was identical to that of CK. This indicates that WT-2 did not fundamentally alter the response rate of erosion intensity to wind speed, but only moderately reduced the amount of erosion at a given wind speed. Once wind speed exceeded 14 m·s^−1^, the protective structure of WT-2 rapidly failed, and erosion amounts surged to levels close to those of CK, suggesting that stubble-only treatments have a critical effective wind speed threshold beyond which standing stubble can no longer effectively disturb airflow and essentially loses its protective function [30]. For WT-3, at low wind speeds, surface straw mulch effectively absorbed wind momentum and weakened near-surface shear stress, providing slightly better protection than WT-1 [31], indicating that surface cover played a dominant role in regulating erosion intensity at this stage. At moderate wind speeds, although T3 lacked standing stubble, the mulch layer alone still maintained a considerable level of protection through its own energy dissipation capacity. At high wind speeds, the advantage of surface straw mulch became evident, with the lowest growth rate of erosion intensity with wind speed (0.12) among all treatments. Even under strong winds of 14–18 m·s^−1^, erosion intensity for WT-3 increased only modestly from 240.20 g·m^−2^·h^−1^ to 470.89 g·m^−2^·h^−1^.

Notably, at the extreme wind speed of 18 m·s^−1^, the anti-wind erosion efficiency of WT-1 still reached 70.15%, significantly outperforming WT-2. This indicates that the combined structure of standing stubble and surface mulching possesses a stronger capacity to regulate intense wind–sand flow, a feature of great importance in wind erosion control. Previous studies have confirmed that a single extreme wind event can cause greater soil loss than the sum of multiple moderate events, and that extreme wind storms are often the key events leading to severe soil erosion [32].

### 5.2. Effects of Different Mulching Treatments on Soil Wind Erosion Amount

The results showed significant differences in field wind erosion amounts under different mulching treatments. Wind erosion amount on the bare peanut field after harvest was significantly greater than that under other treatments, indicating that straw mulching after harvest can effectively suppress wind erosion. For maize fields, the stubble-retained plus mulching treatment exhibited stronger anti-wind erosion capacity than the stubble-only treatment, with net wind deposition observed under the medium-stubble with mulching condition. These results are closely related to the regulatory effects of different mulching treatments on near-surface wind speed, aerodynamic roughness, and soil water content. Peanut harvesting disturbed the soil and destroyed surface protection [33]. During the winter–spring period, the lack of surface cover resulted in low aerodynamic roughness and increased soil water evaporation. The exposed and dry soil surface offered minimal resistance to airflow, making soil particles highly susceptible to wind erosion, which explains why the bare peanut field exhibited the most severe wind erosion [34]. Under the stubble-retained plus mulching mode, standing stubble and surface straw mulch formed a three-dimensional erosion control structure. On the one hand, this configuration significantly increased aerodynamic roughness, directly reduced near-surface wind speed, weakened the driving force of wind on soil particles, and reduced particle entrainment and transport, thereby effectively suppressing wind erosion [35,36]. On the other hand, the surface straw mulch may intercept suspended dust particles in the air and saltating grains near the surface, promoting the fixation of soil particles and reducing wind erosion, or even resulting in net deposition [37]. In contrast, although the stubble-only treatment exhibited a certain erosion reduction effect, it lacked the synergistic effect of surface straw mulching. This resulted in limited capacity to attenuate near-surface wind speed and weaker anti-wind erosion performance compared with the stubble-retained plus mulching treatment [38]. For the intercropped peanut bare land after harvest, straw mulching increased aerodynamic roughness, reduced soil exposure, and raised the threshold wind velocity required for wind erosion initiation, thereby enhancing the soil’s resistance to wind erosion [39]. Furthermore, straw mulching minimized surface soil water loss and increased soil water content before spring sowing, which enhanced the cohesive forces among soil particles and consequently inhibited soil wind erosion [40,41].

Soil wind erosion amounts also differed among mulching treatments with different stubble heights. Notably, the T3 treatment (maize medium stubble with mulching) exhibited a net deposition effect (i.e., net soil accumulation), which may be attributed to the optimal wind erosion control achieved through an appropriate combination of stubble height and straw mulch quantity. Studies have shown that both stubble height and straw mulch quantity significantly affect soil wind erosion amount, with the effect of straw mulch quantity being greater than that of stubble height [42]. Moreover, stubble height influences the amount of straw returned to the field. After the target stubble height was set at harvest, the maize stalks above that height were mechanically shredded and returned to the soil surface as mulch, which caused variations in straw cover percentage among the treatments [43]. The measured straw cover in T3 was higher than that in T1 and T5, possibly due to differences in the contribution of stalks and leaves to the returned residue. Specifically, when the stubble was left excessively high, the amount of residue available for return decreased, resulting in a lower surface straw cover. In addition, surface straw cover was also influenced by the growth conditions of maize in the field. For example, if maize growth in the T1 treatment was poor, aboveground biomass accumulation would be insufficient. Even though the stubble was left relatively low, the total amount of straw available for shredding and returning would still be limited [44]. As a result, the straw cover percentage in T1 could potentially be lower than that in T3. In this study, the surface roughness of low- and medium-stubble plots with mulch was higher than that of high-stubble plots with mulch. Since surface roughness was positively correlated with wind speed attenuation rate, the low- and medium-stubble treatments exhibited stronger wind speed attenuation at 5 cm above the ground than the high-stubble treatment. At this stage, straw cover was the main factor controlling surface roughness and wind erosion. The low- and medium-stubble mulched treatments had more straw cover, so they had an advantage in the early period of erosion control.

However, over time, surface mulch materials are susceptible to displacement and decomposition caused by strong winds, snow cover, and field operations [45,46,47], with residue cover reaching its minimum level by spring sowing (April). The reduction in surface cover altered aerodynamic roughness and near-surface wind speed attenuation capacity [48], resulting in significant differences among stubble heights. Medium-stubble mulched plots exhibited significantly higher aerodynamic roughness than low- and high-stubble mulched plots before spring sowing. Standing stubble in the field can intercept surface mulch to some extent; however, low stubble, due to its short height, has limited capacity to retain straw mulch, resulting in lower aerodynamic roughness by spring sowing and loss of its initial advantage. Studies have shown that when maize stubble height is below 30 cm, the interception rate of wind-eroded particles decreases sharply [49]. High stubble, despite its greater height, suffers from insufficient cover per unit area, which partly weakens its wind erosion control effectiveness. It is worth noting that the medium-stubble plot with mulch (T3) had the highest surface soil water content before spring sowing, which was significantly higher than that of the low-stubble (T1) and high-stubble (T5) plots with mulch. Correlation analysis showed that surface roughness before spring sowing was significantly and positively correlated with soil water content at the same period. The high soil water content in T3 increased the threshold wind speed for soil particle movement. In addition, higher soil water content was favorable for the formation of soil macro-aggregates, which further helped to reduce soil wind erosion [50].

In summary, the medium-stubble treatment with mulch (T3) achieved the highest surface roughness and the highest soil water content during the spring wind erosion period. Regression analysis showed that the wind speed reduction rate of T3 turned positive at a wind speed as low as 0.96 m·s^−1^, which was the lowest threshold among all treatments. This means that T3 could start to reduce wind erosion effectively even at very low wind speeds, and its protective effect remained stable under high wind speeds. This finding is particularly important for the black soil region of Northeast China, where low-speed but long-lasting winds are common in winter and spring. In addition, the reasonable stubble height and straw cover amount in T3 may also trap suspended dust particles and saltating sand grains through physical interception. As a result, some wind-borne materials could be deposited within the field. This eventually led to a net deposition effect in T3, with a wind erosion amount of −0.35 g. Therefore, after the harvest of maize–peanut intercropping fields in the black soil region of Northeast China, applying maize straw mulching to the peanut strips (T7) and medium stubble retention (40–50 cm) plus mulching to the maize strips (T3) can serve as important measures for reducing farmland wind erosion.

It is important to note that the monocropped peanut bare land was used as the control (CK) in this study. Although this approach provided the closest approximation to a bare baseline, it still differed from the actual bare soil conditions of the peanut strips within the intercropping system. In the pre-experiment of the 2024–2025 season, T3 showed a wind erosion amount of −5.45 g, which was also negative, consistent with the −0.35 g observed in the present study. This indicates that the net deposition trend is reproducible to a certain extent. Nevertheless, given the relatively small magnitude of the negative values, it remains difficult to determine whether they represent a genuine net deposition effect or are influenced by measurement uncertainty. It should also be noted that in this study, T7 and T3 were two independent plots within the intercropping system rather than a combined treatment. Therefore, the recommended T7 + T3 scheme is an extrapolation based on the results of two separate treatments, and its actual effect after combination still requires field validation through joint treatment trials. In subsequent research, we intend to conduct 3 to 5 years of continuous in situ monitoring, together with a systematic evaluation of measurement uncertainty. This will allow us to further clarify the differences between the monocropped bare land and the bare land within the intercropping system, and to better understand the causes of the observed negative values, thereby providing a more reliable basis for evaluating the wind erosion control effectiveness of stubble and mulching practices.

## 6. Conclusions

In the wind tunnel simulation experiment, the wind erosion intensity of all treatments increased exponentially with wind speed (R^2^ = 0.90–0.96, *p* < 0.001). Across the entire wind speed range, wind erosion intensity was generally higher in CK and WT-2 than in WT-3 and WT-1. The three mulching treatments showed different protective advantages at different wind speed intervals. At low wind speeds, surface mulch played the primary role in protection, quickly reducing wind erosion forces near the ground. As wind speed increased, the aerodynamic effect of standing stubble became stronger. At high wind speeds, the continuous energy dissipation of surface mulch still dominated the protection, and its performance was more stable than that of stubble alone. Among them, WT-1 (stubble with mulch) maintained an anti-wind erosion efficiency of 70.15% even at the extreme wind speed of 18 m·s^−1^, which was 38.78% higher than that of WT-2 (stubble without mulch). This indicates that the combination of stubble and mulch provides a significant synergistic advantage in erosion control under high wind speeds.

Field wind erosion amounts ranked as CK (67.53 g) > T6 (29.00 g) > T2 (27.09 g) > T4 (23.53 g) > T1 (16.18 g) > T7 (9.87 g) > T5 (3.80 g) > T3 (−0.35 g). Under the same stubble height, the wind erosion amount of stubble-with-mulch treatments was lower than that of stubble-without-mulch treatments. Among different stubble heights, the medium-stubble treatment with mulch (T3) showed a net deposition effect, with a reduction of 100.5% compared with CK, representing the best performance in erosion control. In this treatment, standing stubble and surface mulching synergistically increased aerodynamic roughness by 29.74–51.47%, enabling effective wind erosion protection at low wind speeds by attenuating near-surface wind speed (attenuation rate of 50.77–72.57%). Meanwhile, straw mulching reduced soil water evaporation, increased pre-sowing surface soil water content (by 126.04%), and enhanced inter-particle cohesion, thereby increasing the threshold wind velocity for wind erosion initiation.

From a temporal dynamics perspective, the low stubble retention with mulching treatment (T1) had an initial advantage after autumn harvest due to its greater straw mulching amount. However, by the pre-sowing period in spring, the aerodynamic roughness of the medium stubble retention with mulching treatment (T3, 2.53 cm) was significantly higher than that of low stubble (T1, 2.24 cm) and high stubble (T5, 2.12 cm), and its soil water content was the highest among all treatments, indicating that medium stubble height (40–50 cm) can maintain higher surface protection capacity during the spring wind erosion peak period. The peanut strip straw mulching treatment (T7), despite the absence of standing stubble, achieved a near-surface (5 cm) wind speed attenuation rate of 62.65% after autumn harvest, with significantly higher surface roughness before spring sowing than other treatments, a 46.22% increase in pre-sowing soil water content, and a wind erosion reduction rate of 85.38%.

In summary, after harvest in the maize–peanut intercropping system in the black soil region of Northeast China, the combined application of maize straw mulching on peanut fields (T7, with a straw mulching quantity of no less than 7800 kg·hm^−2^) and maize stubble retention at medium height (40–50 cm) combined with straw mulching on maize fields (T3, with a straw mulching quantity of no less than 6900 kg·hm^−2^) can effectively suppress wind erosion in maize–peanut intercropping farmland, with the potential to even induce net wind deposition. These two practices are recommended as preferred technical options for post-harvest wind erosion control in the study area and similar brown soil regions susceptible to wind erosion.

## Figures and Tables

**Table 1:** Design of wind tunnel experimental treatments.

Treatment	Land Type	Soil Surface Conditions	Straw Mulch Quantity (kg·hm^−2^)
CK	Monocropped peanut harvested bare land	Spring-sown peanut field left as winter fallow after winter plowing	0
WT-1	Intercropped maize stubble with mulching	Stubble retention + straw mulching, stubble height 50 cm	7250.4
WT-2	Intercropped maize stubble without mulching	Stubble only without mulching, stubble height 50 cm	0
WT-3	Intercropped peanut with mulching, no stubble	Straw mulching only, no standing stubble	8313.6

**Table 2:** Design of wind erosion cycle experimental treatments.

Treatment	Land Type	Soil Surface Conditions	Straw Mulch Quantity (kg·hm^−2^)
CK	Monocropped peanut harvested bare land	Spring-sown peanut field left as winter fallow after winter plowing	0
T1	Intercropped maize with low stubble + mulching	Stubble retention + mulching, stubble height 10–20 cm	6857.4
T2	Intercropped maize with low stubble, no mulching	Stubble retention only, no mulching, stubble height 10–20 cm	0
T3	Intercropped maize with medium stubble + mulching	Stubble retention + mulching, stubble height 40–50 cm	6925.1
T4	Intercropped maize with medium stubble, no mulching	Stubble retention only, no mulching, stubble height 40–50 cm	0
T5	Intercropped maize with high stubble + mulching	Stubble retention + mulching, stubble height 60–70 cm	6270.3
T6	Intercropped maize with high stubble, no mulching	Stubble retention only, no mulching, stubble height 60–70 cm	0
T7	Intercropped peanut with mulching, no stubble	Straw mulching only, no standing stubble	7885.4

**Table 3:** Curve fitting equations for soil wind erosion intensity (*Q*) versus wind speed (*V*).

Treatment	Curve-Fitting Equation	*R* ^2^	*p*	RMSE
CK	*Q* = 91.39 e^0.15*V*^	0.96	<0.001	109.85
WT-1	*Q* = 40.92 e^0.13*V*^	0.90	<0.001	42.55
WT-2	*Q* = 65.40 e^0.15*V*^	0.92	<0.001	94.11
WT-3	*Q* = 48.62 e^0.12*V*^	0.93	<0.001	38.52

**Table 4:** Regression equations for the relationship between wind speed attenuation rate (*Y*) and near-surface wind speed (*V*).

Treatment	Regression Equations	*R* ^2^	*p*	*RMSE*	*n*
T1	*Y* = −123.00 + 140.18 *V* − 26.92 *V*^2^	0.93	0.001	12.64	277
T2	*Y* = −25.00 + 70.86 ln(*V* − 0.49)	0.99	<0.001	13.45	158
T3	*Y =* 52.51 + 14.38 ln(*V* − 0.95)	0.98	<0.001	3.17	215
T4	*Y* = 33.85 + 23.06 ln(*V* − 0.79)	0.97	<0.001	2.72	202
T5	*Y* = −132.01 + 160.43 ln(*V* + 0.25)	0.96	<0.001	13.97	224
T6	*Y* = −25.45 + 85.68 ln(*V* − 0.14)	0.99	<0.001	4.59	189
T7	*Y* = 67.54,V<3.52146.11−1233.79 V+227.85V2−13.71V3,V≥3.5	0.98	0.004	7.54	196

## Data Availability

The original contributions presented in the study are included in the article. Further inquiries can be directed to the corresponding author.
